# Influence of Phlai (*Zingiber montanum*) and Njui (*Bombax ceiba*) Extracts in Bull Semen Extender on Antioxidant Activity and Sperm Quality

**DOI:** 10.3390/molecules31020368

**Published:** 2026-01-20

**Authors:** Jiraporn Laoung-on, Nopparuj Outaitaveep, Jakree Jitjumnong, Sakaewan Ounjaijean, Kongsak Boonyapranai

**Affiliations:** 1 Research Institute for Health Sciences, Chiang Mai University, Chiang Mai 50200, Thailand; jiraporn.l@cmu.ac.th (J.L.-o.); nop.outaitaveep@gmail.com (N.O.); sakaewan.o@cmu.ac.th (S.O.); 2 Office of Research Administration, Chiang Mai University, Chiang Mai 50200, Thailand; 3 Department of Animal and Aquatic Sciences, Faculty of Agriculture, Chiang Mai University, Chiang Mai 50200, Thailand; jakree.j@cmu.ac.th

**Keywords:** sperm motility, phytochemicals, CASA, ROS, male reproductive system

## Abstract

Infertility represents a significant global health issue, and the use of antioxidants in sperm preservation techniques provides an effective strategy to improve sperm quality. This study aims to examine the phytochemical components of Phlai and Njui extracts and their antioxidant effects on enhancing the motility of fresh bull semen. Among the extracts, Njui contained the highest levels of total phenolics, total tannins, and lycopene contents along with the strongest DPPH, ABTS, and AOPP inhibition. Phlai contained the highest levels of total flavonoids. Njui and combined extracts showed the strongest AGE inhibition. The motility of sperm in the semen extender supplemented with Phlai, Njui, and their combination exhibited greater total motility, particularly progressive motility, compared to sperm in the normal extender after 48–72 h. Furthermore, there was a reduced generation of ROS compared to sperm in the normal extender and with vitamin E acetate supplementation after 24–72 h. In conclusion, Phlai and Njui extracts, plentiful in bioactive chemicals, showed significant antioxidant activity and enhanced sperm motility by neutralizing free radicals and strengthening antioxidant defenses. The findings indicate that Phlai and Njui, especially in combination, provide advantages for sperm preservation.

## 1. Introduction

Male reproductive dysfunction is a significant contributor to infertility, which has considerable implications for population growth [1]. Globally, half of infertility cases are caused by male factors, which are related to sperm parameters [2]. An imbalance between reactive oxygen species (ROS) and the antioxidant defense system leads to cellular damage, recognized as a significant contributor to male infertility [3].

Lipid peroxidation, protein oxidation, and DNA damage have been demonstrated to impair spermatogenesis, resulting in decreased sperm concentration, motility, viability, and normal morphology [4,5,6,7]. The characteristics of sperm quality are essential factors influencing male reproductive capacity [5,6]. Oxidative stress has been demonstrated to reduce fertilization potential, thereby contributing to male infertility [3]. As a result, the application of assisted reproductive technologies (ARTs) in humans and animals has risen to enhance reproductive achievements [8]. Sperm quality is a crucial determinant of procedural success across these applications.

Before artificial insemination, semen samples are gathered and processed to isolate high-quality spermatozoa for preservation and future application [9]. Semen extenders are essential for preserving spermatozoa during storage and cryopreservation by guarding cellular structures and reducing oxidative stress linked to freezing and natural deterioration [9,10]. A previous study has demonstrated that forward progressive motility is a crucial factor in fertilization success [2,11], and high-quality extenders facilitate effective sperm movement towards the oocyte [12]. Nonetheless, the success rates of artificial insemination of bulls remain relatively low, approximately 40–50% in 2021 [13], thereby increasing operational expenses and constraining reproductive efficiency.

Male reproductive performance may be enhanced by reducing oxidative stress, with antioxidant consumption acting as an essential protective factor [3]. Synthetic antioxidants, as effective, are costly and have been associated with substantial deleterious effects [14], including disruption of DNA synthesis, carcinogenic potential, fibrosis, and embryotoxicity [15]. Consequently, natural antioxidants have received increased popularity as safer and more sustainable options for reducing oxidative stress.

Phlai (*Zingiber montanum*: *Z. montanum*) is a Thai medicinal plant recognized as an essential herbal ingredient by the National List of Essential Medicines, Ministry of Public Health, Thailand. Phlai has been extensively utilized in traditional medicine, and prior research has shown that it contains essential phytochemicals with anti-inflammatory, antioxidant, and antibacterial activities [16,17]. Njui (*Bombax ceiba* L.: *B. ceiba*) is a remarkable flowering tree from the Bombacaceae family, demonstrating several biological properties such as anti-inflammatory, aphrodisiac, antibacterial, hepatoprotective, anti-diabetic, anti-aging, and hypotensive benefits [18,19,20,21]. The stamen of Njui is acknowledged as a traditional medicinal ingredient with significant economic value, but the petals are typically considered agricultural byproducts. Research on the biological characteristics of Njui petals is currently limited.

Furthermore, the potential use of Phlai, Njui petals, and their combined extracts in enhancing sperm resilience and improving sperm preservation in semen extender for artificial insemination has not been thoroughly explored. Accordingly, the present study aimed to investigate the effects of Phlai and Njui petal extracts supplemented in bull semen extenders or diluents on antioxidant potential and sperm motility, with the objective of reducing the costs associated with artificial insemination, improving fertilization success rates, and providing insights for future approaches in human sperm preservation.

## 2. Results

### 2.1. Detection and Measurement of Phytochemical Constituents

The levels of total phenolics, tannins, flavonoids, monomeric anthocyanins, and lycopene of Phlai rhizome and Njui petal are presented in Table 1. Njui extract had significantly the highest total phenolic and total tannin contents, followed by the mixture of Phlai and Njui, and the Phlai extract. In contrast, the total flavonoid content was significantly highest in the Phlai extract, followed by the mixture and the Njui extract. Pure Njui and the mixture had significantly higher lycopene content than the Phlai extract. Total monomeric anthocyanins were not detected in any of the extracts (Table 1).

Representative HPLC chromatograms of the Phlai extract are displayed in Figure S1. Quantification was carried out by comparing the peak areas of sample constituents with those of standard compounds of known concentrations. The Phlai extract contained significantly higher amounts of capsaicin, apigenin, kaempferol, and quercetin than the mixture and the Njui extract. Apigenin, kaempferol, and quercetin were not detected in the Njui extract. However, the Njui extract significantly showed the highest concentration of gallic acid, chlorogenic acid, ellagic acid, and rutin, followed by the mixture extract and the Phlai extract (Table 2).

### 2.2. Antioxidant and Anti-Glycation Capacities in Cell-Free System

**Figure 1:**
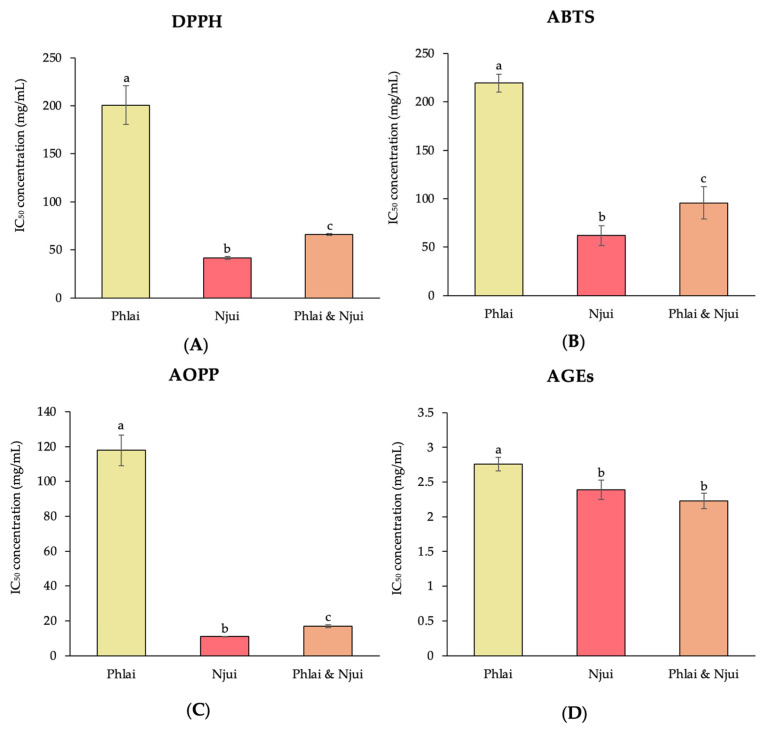
The half-maximal inhibitory concentration of DPPH (**A**), ABTS (**B**), AOPP (**C**), and AGEs (**D**) of the Phlai, the Njui, and the mixture of Phlai and Njui extracts are presented as mean value ± SEM (error bars). Data were collected from three replications (n = 3). Different superscript letters indicate significant differences between groups at *p* < 0.05. All parameters were analyzed by one-way ANOVA followed by Duncan’s test.

The half-maximal inhibitory concentrations (IC_50_) for 2,2-diphenyl-1-picrylhydrazyl (DPPH), 2,2′-azino-bis (3-ethylbenzothiazoline-6-sulfonic acid) (ABTS), and advanced oxidation protein product (AOPP) inhibition were significantly lower for the Njui extract compared with the Phlai and mixed extracts (Figure 1A–C). Additionally, the Njui and mixed extracts exhibited significantly lower IC_50_ values for the inhibition of advanced glycation end products (AGEs) than the Phlai extract (Figure 1D).

### 2.3. Cytotoxicity Determination

**Figure 2:**
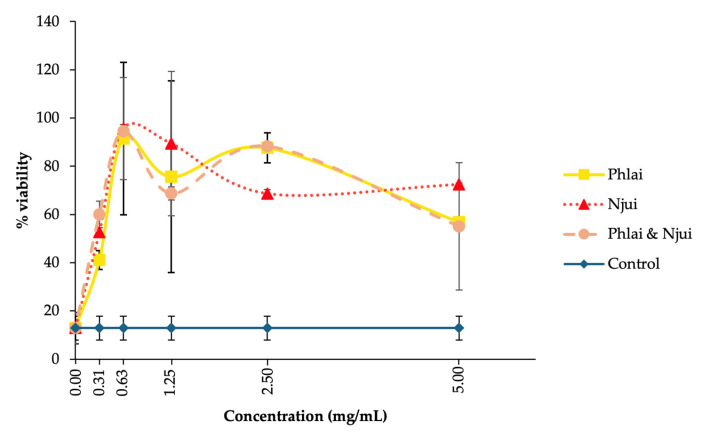
Sperm viability (%) of bull semen treatment with Phlai, Njui, and combined extracts at concentrations of 0.31, 0.63, 1.25, 2.5, and 5 mg/mL compared with the normal control. Data were collected from three replications (n = 3). Values are presented as mean ± SEM (error bars).

An MTT assay (3-[4,5-dimethylthiazol-2-yl]-2,5-diphenyl tetrazolium bromide) was performed to assess cytotoxicity in bull sperm and to determine the appropriate extract concentration for subsequent sperm quality analyses. Among the normal control groups treated with Phlai, Njui, and the combined extracts, the 0.63 mg/mL dose yielded the highest sperm viability after 3 h of incubation at 37 °C, with viability rates of 91.49%, 95.63%, and 94.48% of the initial values, respectively (Figure 2). These treatments demonstrated improved sperm preservation compared to the untreated control, which exhibited a viability reduction to 12.87% from the beginning of the experiment.

### 2.4. Sperm Motility

The proportion of local motility (LM), the sperm movement restricted to a small area without forward progression, was significantly reduced in the extender supplemented with vitamin E acetate and Phlai extract after 24 h of incubation at 4–5 °C compared with the normal extender. Furthermore, after 48 h of incubation, the extender containing the combined extracts showed a significant increase in progressive motility (PM) relative to the normal extender, while sperm immotility (IM) was significantly decreased compared with the control. In addition, total motility (TM) and PM were significantly enhanced in extenders containing Phlai, Njui, or the combined extracts, whereas local motility (LM) and IM were significantly reduced compared with the normal control (Table 3).

### 2.5. Reactive Oxygen Species Levels in Spermatozoa

**Figure 3:**
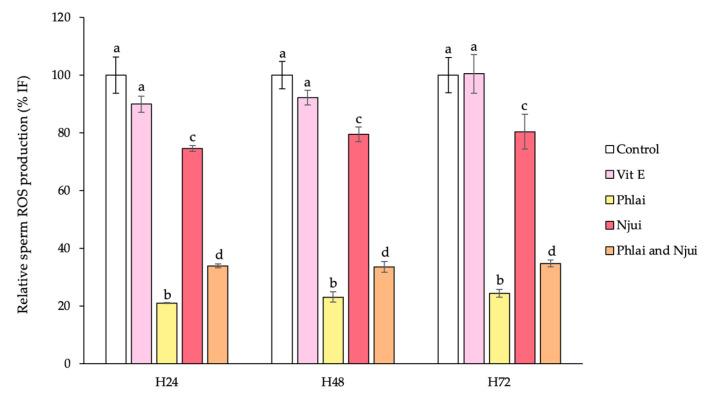
Mean ± SEM (with error bars) of relative sperm ROS production in fresh bull sperm samples extended under the following conditions: control, Vitamin E acetate (Vit E), Phlai, Njui, and the combined extract after incubation at 4–5 °C for 24, 48, and 72 h. Data were derived from three independent replicates (n = 3). Different superscript letters indicate significant differences between groups at *p* < 0.05. The %IF (fluorescence intensity) indicates the percentage of fluorescence intensity relative to the control extender, representing the level of ROS activity in sperm cells. All parameters were analyzed by one-way ANOVA followed by Duncan’s test.

**Figure 4:**
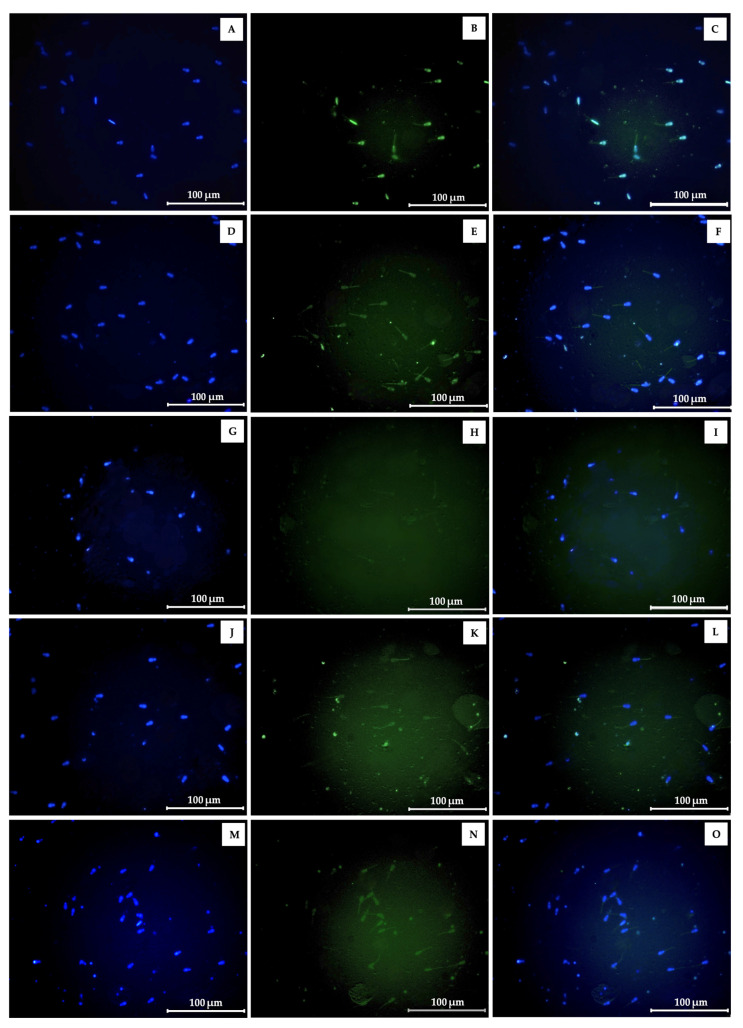
General morphological appearance and ROS fluorescence intensity of cattle sperm in the normal (**A**–**C**), vitamin E acetate (**D**–**F**), Phlai (**G**–**I**), Njui (**J**–**L**), and the combined (**M**–**O**) extenders after 24 h, stained with DCFH-DA (DCFH-DA: 2′,7′-dichlorodihydrofluorescein diacetate/DAPI (4′,6-Diamidino-2-Phenylindole, Dihydrochloride) and observed at 400× magnification under a fluorescence microscope (Zeiss, Oberkochen, Germany). Sperm stained blue with DAPI represent the total sperm population, whereas those emitting green fluorescence after DCFH-DA staining indicate ROS-producing sperm. The merged image showing both blue and green signals is presented in the right column.

The relative ROS production in bovine spermatozoa preserved in various extender formulations at 24, 48, and 72 h is shown in Figure 3. Sperm samples incubated in the extender with Phlai, Njui, and the combined extracts exhibited significantly lower ROS levels compared to those in the standard egg-yolk extender and the extender supplemented with vitamin E acetate across all time points. Additionally, the morphological appearance and ROS fluorescence intensity of sperm in the normal extender after 24 h of incubation are illustrated in Figure 4.

## 3. Discussion

This study employed ultrasonic extraction, a modern technique that utilizes ultrasound energy together with suitable solvents to efficiently isolate target compounds from natural products [22]. The application of ultrasound improves solvent penetration and mass transfer, thereby reducing extraction time, lowering energy requirements, and minimizing the temperatures needed during the extraction process [23]. The Njui extract contained the highest amount of total phenolics, total tannins, and lycopene contents, followed by the combined and the Phlai extracts, respectively. Similarly, previous studies have shown that the flowers and stamens of Njui (*B. ceiba*) contain high levels of total phenolic and total tannin contents [24,25,26]. However, when using the same extraction method, the Njui petal extract exhibited higher concentrations of phenolics and tannins than the stamen extract. Nevertheless, the Phlai extract contained the highest amount of total flavonoid content, followed by the combined and Njui extracts. Flavonoids, especially members of the flavone and flavanol subclasses, are commonly responsible for yellow coloration in plant tissues, as they exhibit characteristic light absorption properties that produce visible yellow hues [27,28]. Therefore, the yellow color of Phlai rhizomes suggests that they contain a higher concentration of flavonoids compared to the red-colored Njui petals.

The HPLC chromatogram of the Njui petal extract revealed the presence of capsaicin, gallic acid, chlorogenic acid, ellagic acid, and rutin, whereas the Phlai extract exhibited capsaicin, gallic acid, chlorogenic acid, ellagic acid, apigenin, kaempferol, rutin, and quercetin. The Phlai extract presented a higher concentration of capsaicin, apigenin, kaempferol, and quercetin compared to the Njui and combined extracts. Capsaicin has been reported to rescue sperm damage in rat models of testicular stress [29]. Apigenin and kaempferol showed protective effects in sperm cryopreservation models, enhancing post-thaw motility and membrane integrity, probably via ROS scavenging and stability of mitochondrial and heat-shock responses [30,31]. Quercetin reliably promotes sperm motility and viability in a dose and time-dependent manner, improving the performance of frozen-thawed sperm [32]. However, the Njui extract showed a higher concentration of gallic acid, chlorogenic acid, ellagic acid, and rutin than the Phlai and combined extracts. Gallic acid and rutin have demonstrated the ability to enhance sperm motility, viability, and steroidogenic characteristics in animal models by restoring antioxidant defenses [33,34]. Chlorogenic and ellagic acids protect testicular tissue and spermatozoa from oxidative damage induced by toxicants through ROS scavenging and inflammatory regulation [35,36]. The pure extract had higher levels of several particular bioactive components, but it did not include some important molecules that are linked to male reproductive function. The combined extract, on the other hand, is a better choice because it has all the right chemicals in the right amounts.

The antioxidant activity of the Phlai, Njui, and combined extracts in a cell-free system was determined using DPPH, ABTS, AOPP, and AGE assays. The Njui extract presented significantly lower IC_50_ concentrations in DPPH, ABTS, and AOPP assays than the Phlai and combined extracts, which indicates higher antioxidant capacity. Consequently, Njui’s enhanced performance indicates a superior capacity to neutralize free radicals and reduce oxidative protein damage compared to the Phlai and combined extracts. The rise in activity is likely due to greater quantities of total phenolics, tannins, and particular phenolic acids (e.g., gallic acid, chlorogenic acid, and ellagic acid), which are well-established for their potent hydrogen-donating and electron-transfer capabilities [37,38,39]. Moreover, both the Njui and combined extracts had significantly lower IC_50_ values for AGE inhibition compared to the Phlai extract, indicating a superior ability to inhibit glycoxidation processes. The production of AGEs contributes to oxidative stress and cellular dysfunction [40]. Thus, the Njui and combined extracts’ capacity to suppress AGE accumulation indicates possible protective effects on biomolecules in oxidative environments.

The MTT findings demonstrate that all tested extracts, including Phlai, Njui, and their combination, showed minimal cytotoxicity towards cattle sperm at the tested concentrations, with the 0.63 mg/mL concentration providing the best viability preservation. Following 3 h of incubation at 37 °C, sperm treated with Njui, Phlai, and combination extracts demonstrated a vitality above 90%, whereas the untreated control demonstrated a significant reduction to 12.87%. The observed protective effects are likely due to the antioxidant and membrane-stabilizing characteristics of the bioactive compounds in the extracts, including phenolics, flavonoids, and other polyphenols, which mitigate oxidative stress and prevent mitochondrial dysfunction, as indicated by the reduction of MTT to formazan in viable sperm [41,42]. Similarly, it had been found that *B. ceiba* stamen possesses significant antioxidant activity and can sustain sperm viability [25,26].

The findings of the current study indicate that plant extracts, individually and in combination, have varying impacts on sperm motility metrics. The significant decrease in local motility (LM) observed after 24 h in extenders supplemented with vitamin E acetate (positive control) and Phlai extract indicated that these treatments may improve the ratio of physiologically relevant progressive movement, as LM is generally linked to non-productive or energy-inefficient sperm activity [43]. Consequently, the reduction in LM suggests that the Phlai extract and vitamin E acetate may aid in preserving mitochondrial function and enhancing flagellar activity [43]. After 48 h of incubation, the combined extract demonstrated a significant enhancement in sperm motility, indicated by a considerable rise in progressive motility (PM) and a corresponding decrease in immotile sperm (IM). Moreover, extenders containing Phlai, Njui, or their combination significantly enhanced total motility (TM) and progressive motility (PM) while reducing local motility (LM) and immotility (IM) compared to the control, as well as the positive control. The evidence suggests that bioactive components, such as phenolics, flavonoids, and tannins, may function separately or synergistically to enhance energy metabolism, improve membrane fluidity, and maintain ionic homeostasis in spermatozoa. Similarly, prior research indicates that the incorporation of plant-derived antioxidant extracts can preserve sperm quality and alleviate oxidative damage under storage conditions [42,44]. Furthermore, the preceding study has shown that *B. ceiba* stamen extract promotes cattle sperm motility and viability under oxidative stress, while also increasing epididymal sperm count, thus supporting its role in stimulating spermatogenesis [25,26,45]. Nevertheless, there is limited direct information regarding the influence of Phlai on sperm function, highlighting a knowledge gap. The coordinated enhancement of motility characteristics indicates a transition to more efficient and physiologically appropriate movement patterns, essential for fertilization potential. Moreover, the supplementation of semen extenders with Phlai, Njui, and their combination effectively reduces ROS production in cattle spermatozoa during incubation, which corresponds with the enhancement of sperm motility. Moreover, ROS production of sperm in Phlai supplementation showed the lowest antioxidant capacity, followed by the combined extract and Njui supplementation, which contrasts with the result of the cell-free antioxidant assay, in which Njui showed the highest antioxidant capacity. The difference in cell-free antioxidant levels and sperm model may be the result of the permeability of bioactive substances through the sperm plasma membrane. Phlai contains more lipophilic flavonoids, such as capsaicin, apigenin, kaempferol, and quercetin, which are known to have a greater membrane permeability [46,47]. In contrast, Njui is mostly composed of gallic acid, chlorogenic acid, ellagic acid, and rutin, which are more hydrophilic, have a higher polarity, and limit membrane transport [48,49]. Consequently, compounds derived from Phlai may infiltrate sperm cells more effectively and have enhanced intracellular ROS-scavenging properties than those from Njui. The reduction in ROS likely contributes to maintaining cell membrane integrity and mitochondrial function [3] by supporting increased total motility (TM) and progressive motility (PM), as well as a concomitant decrease in local motility (LM) and immobile spermatozoa (IM). These findings collectively demonstrate the efficacy of herbal extracts, especially in combination, as functional additives in semen extenders to enhance sperm quality and maintain motility during storage. Although this study shows positive results from using the extracts for preserving antioxidant properties and sperm motility in vitro, it is possible that this extract may exert its mechanism of action on sperm, which may affect sperm fertilization. Moreover, although the Njui extract showed strong antioxidant activity in cell-free system, its lower permeability across the sperm membrane may limit its intracellular effect, suggesting that future studies should explore formulation improvements, such as encapsulation, to enhance its biological efficacy. Additionally, the experiments were conducted using semen collected from a limited number of bulls, and the findings were derived from in vitro evaluations only. Further studies involving a larger number of bulls, as well as validation though in vivo fertility trials, are necessary to confirm the practical applicability of the proposed extender formulation.

## 4. Materials and Methods

### 4.1. Collection and Preparation of Plant Extracts

**Figure 5:**
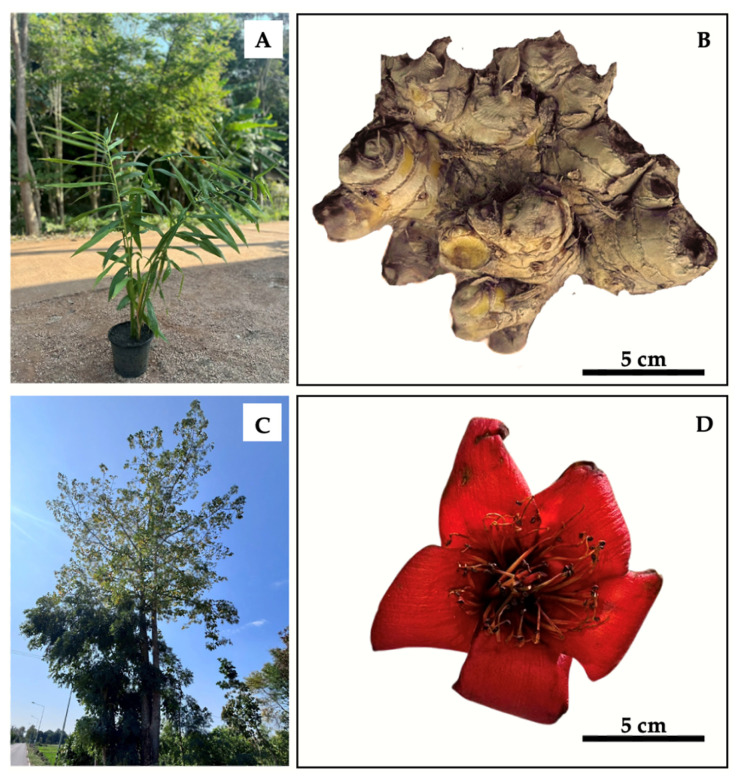
General appearance of the Phlai tree (**A**), Phlai rhizome (**B**), Njui tree (**C**), and Njui flower (**D**).

Fresh rhizomes of Phlai (*Zingiber montanum*), approximately three years old, were purchased from a local herbal market in the Hot district, Chiang Mai province, Thailand. In addition, the petals of Njui (*Bombax ceiba*) in the red variety were collected from an open field in the lower northern region of Thailand (Laplae district, Uttaradit Province, Thailand). The Phlai rhizomes and Njui petals were thoroughly washed, sliced, and dried using a heat plate. The dried samples were then dehumidified in a hot-air oven at 40 °C for 24 h and pulverized into a fine powder. The powders were extracted using the ultrasonication method, and the extract was subsequently filtered, lyophilized, and stored at −20 °C until use in antioxidant and sperm quality assessment. Phlai and Njui samples were deposited and authenticated at the Herbarium, Faculty of Pharmacy, Chiang Mai University, under the voucher specimen numbers 0023423 and 0223382, respectively. The Phlai tree, Phlai rhizome, Njui tree, and Njui flower are demonstrated in Figure 5.

### 4.2. Determination of Total Phenolic and Total Tannin Content

The total phenolic and tannin contents of Phlai and Njui extracts were analyzed using the Folin–Ciocalteu colorimetric assay, with slight modifications based on previously described methods [26]. For the determination of total phenolics, 100 µL of each sample (1 mg/mL) was combined with 500 µL of 10% Folin–Ciocalteu reagent and 400 µL of 1 M sodium carbonate (Na_2_CO_3_). The mixture was left to react for 15 min at room temperature, after which the absorbance was recorded at 765 nm using a spectrophotometer (BMG LABTECH, Ortenberg, Germany). Gallic acid served as the standard, and the results are expressed as micrograms of gallic acid equivalents (µg GAE) per gram of plant extract.

Similarly, total tannin content was quantified using the same reagent system, where 100 µL of each sample (1 mg/mL) was mixed with 500 µL of 10% Folin–Ciocalteu reagent and 400 µL of 1 M sodium carbonate (Na_2_CO_3_). The reaction mixture was incubated for 30 min at room temperature, and the absorbance was measured at 700 nm. Tannic acid was used to construct the calibration curve, and the concentration is expressed as micrograms of tannic acid equivalents (µg TAE) per gram of plant extract.

### 4.3. Determination of Total Flavonoid Content

The total flavonoid content was analyzed using a colorimetric method. In summary, 100 µL of the plant extract was mixed with 50 µL of 10% aluminum chloride solution and allowed to stand for 30 min at room temperature. Subsequently, 50 µL of potassium acetate and 700 µL of distilled water were added to the mixture. The absorbance was then recorded at 415 nm using a spectrophotometer. The results are expressed as micrograms of quercetin equivalents (µg QE) per gram of plant extract.

### 4.4. Determination of Total Anthocyanins

The total monomeric anthocyanin content in the plant extracts was quantified using the pH differential spectrophotometric method. Two buffer systems were employed for analysis: 0.025 M potassium chloride (pH 1.0) and 0.4 M sodium acetate (pH 4.5). For each condition, 100 µL of the sample extract was combined with 900 µL of the respective buffer solution in a 5 mL test tube. The mixtures were allowed to stand at room temperature for 15 min. The concentration of monomeric anthocyanins was calculated according to the following equation:

Monomeric anthocyanin pigment (µg/mL) = (A × MW × DF × 1000)/(ε × 1)

where A represents the absorbance difference between pH 1.0 and pH 4.5, MW is the molecular weight of cyanidin-3-glucoside (449.2 g/mol), and ε denotes the molar absorptivity coefficient (26,900 L·mol^−1^·cm^−1^). The results are expressed as micrograms of cyanidin-3-glucoside equivalents (µg C3G) per gram of plant extract.

### 4.5. Determination of Lycopene Content

The lycopene content of Phlai and Njui samples was quantified using a hexane–ethanol–acetone mixture (2:1:1, *v*/*v*). Briefly, 1 g of dried sample was mixed with 1 mL of distilled water, vortexed, and incubated at 30 °C for 60 min. Then, 8 mL of the solvent mixture was added, vortexed, and incubated in the dark for 10 min. After adding 1 mL of distilled water and vortexing again, the supernatant was collected, and absorbance was measured at 503 nm using a spectrophotometer. Lycopene content (mg/g dry weight) was calculated using the following equation:

Lycopene = (A_503_ × 537 × 8 × 0.55)/(0.10 × 172).

### 4.6. Phytochemical Compound Analysis by High-Performance Liquid Chromatography (HPLC)

The phytochemical composition of Phlai and Njui extracts was analyzed using high-performance liquid chromatography (HPLC) coupled with a diode array detector (Shimadzu SIL-20AC Prominence Autosampler, Shimadzu, Tokyo, Japan) [26]. Separation was performed on a Purospher^®^ Star PR-18 (Agilent 1260 Infinity Binary LC, Santa Clara, CA, USA) endcapped column (150 × 4.6 mm, 5 µm). The mobile phase consisted of solvent A (0.1% formic acid in water, 92%) and solvent B (acetonitrile, 8%), maintained for 10 min. The proportion of solvent B was then increased to 14% at 24 min, 23% at 35 min, and 24% at 60 min. A 20 µL aliquot of the Phlai sample was injected, and detection was carried out at 250, 330, and 360 nm [50], with spectra recorded between 200 and 400 nm. Compound identification was based on comparison of retention times and UV–Vis spectral profiles with those of reference standards. The concentrations of capsaicin, gallic acid, chlorogenic acid, ellagic acid, apigenin, kaempferol, rutin, and quercetin were calculated using the external calibration curves with coefficients of determination (R^2^) ≥ 0.99.

### 4.7. Determination of Antioxidant Properties

#### 4.7.1. Assay of 2,2-Diphenyl-1-picrylhydrazyl (DPPH) Radical Scavenging Activity

The antioxidant potential of Phlai and Njui extracts was assessed using the DPPH free radical scavenging assay. Briefly, 50 µL of each extract at different concentrations was mixed with 200 µL of 0.004% DPPH solution prepared in methanol. Vitamin E acetate was used as a reference antioxidant. The reaction mixtures were incubated in the dark at room temperature for 30 min, after which absorbance was recorded at 515 nm using a microplate reader. The half-maximal inhibitory concentration (IC_50_) values were subsequently calculated.

#### 4.7.2. Assay of 2,2′-Azino-di-3-ethylbenzthiazoline Sulfonate (ABTS) Radical Scavenging Activity

The ABTS assay was employed to determine the free radical scavenging activity of Phlai and Njui extracts. A 7 mM ABTS stock solution was prepared in distilled water and kept at 4 °C in the dark. The solution was subsequently diluted to obtain an absorbance of 0.7 at 734 nm. Then, 50 µL of each extract at various concentrations was mixed with 200 µL of the ABTS working solution and incubated at room temperature for 30 min in the dark. Absorbance was recorded at 515 nm using a microplate reader, with vitamin E acetate used as the standard antioxidant. The IC_50_ value of the extract was then determined.

#### 4.7.3. Advanced Oxidation Protein Products (AOPP) Inhibition Assay

Initially, 50 µL of each extract at various concentrations was mixed with 100 µL of bovine serum albumin (BSA, 1 mg/mL) prepared in 0.2 M phosphate-buffered saline (PBS, pH 7.4). Protein oxidation was initiated by adding 15 µL of 0.07 M FeSO_4_. The mixtures were incubated at room temperature for 30 min in the dark. Subsequently, 50 µL of 1.16 M potassium iodide (KI) was added, followed by a 2 min incubation. Absorbance was measured at 340 nm using a microplate reader, and the half-maximal inhibitory concentration (IC_50_) values were calculated.

### 4.8. Advanced Glycation End Product (AGE) Inhibition Assay

The inhibitory effect of Phlai and Njui extracts on advanced glycation end-products (AGEs) was assessed following a previously reported method. Briefly, 50 µL of each extract at varying concentrations was combined with 50 µL of BSA, and glycation was induced by adding 50 µL of 1 M D-glucose. The mixtures were incubated at 50 °C for 24 h in the dark. Fluorescence intensity was measured using a microplate reader (Perkin Elmer, Singapore) at an excitation wavelength of 360 nm and an emission wavelength of 460 nm, and the half-maximal inhibitory concentration (IC_50_) was calculated.

### 4.9. Cytotoxicity Determination

Cytotoxicity was first conducted using frozen-thawed semen as a preliminary screening to evaluate the appropriate dose. This strategy enabled the selection of suitable doses before the development and determination of the extender in the fresh semen. Employing frozen semen at this stage helped to reduce the use of animals and experimental interventions, in line with ethical considerations in animal studies.

Frozen Charolais bull semen straws were obtained from Namchuea Wongwi Company Ltd. (Chiang Mai, Thailand). The semen was thawed and centrifuged at 2500 rpm for 5 min. The resulting sperm pellet was washed three times with Krebs medium (pH 7.4) and adjusted to a concentration of 10 × 10^6^ sperm/mL. Aliquots of 100 µL sperm suspension in 96-well plates were treated with five concentrations (0.31, 0.63, 1.25, 2.5, and 5 mg/mL) of Phlai and Njui extracts. The samples were incubated at 37 °C for 3 h, after which sperm viability was assessed using the MTT assay. Viability was expressed as a percentage relative to the control, and selected concentrations were used for fresh semen extender evaluation.

### 4.10. Animal and Ethical Approval

Fresh semen samples were obtained from one white Lumphun bull (*Bos indicus*), aged 7 years old and weighing 550–600 kg. The animals were confirmed to be clinically healthy, with normal reproductive organs and satisfactory libido. They were maintained individually in controlled conditions at 22–27 °C. The White Lumphun cattle were obtained from the Department of Animal and Aquatic Science, Faculty of Agriculture, Chiang Mai University, Chiang Mai, Thailand. The experimental procedure was approved by the Animal Ethics Committee, Faculty of Agriculture, Chiang Mai University (No. AG1007/2567) and agreed with the institutional guides for the Animal Care and Use.

### 4.11. Semen Collection

Fresh semen collection was performed using a rubber artificial vagina (AV). A bull was stimulated by teaser cows. When the white Lumphun bull showed mounting behavior toward the teaser female, the prepuce was held, and the penis was guided into the AV during ejaculation. After ejaculation, the AV was detached and either examined immediately in the laboratory or temporarily stored at 5 °C in a thermostat. To prevent light-induced damage, a dark-colored AV was used. Additionally, pre-warmed glass bottles were utilized for semen collection. Two successive ejaculates were obtained weekly for three consecutive weeks. Immediately after collection, the semen samples were transported to the laboratory and maintained in a water bath at 33–34 °C. The semen was first evaluated macroscopically for volume and concentration, followed by a microscopic assessment of mass motility, individual motility, and morphological abnormalities. Only fresh semen samples with motility greater than 80% and abnormalities below 15% were selected for inclusion in this study.

### 4.12. Extender Preparation

Fresh semen samples that met the specified quality criteria were subjected to the dilution process. Dilution was performed using an egg yolk–Tris extender composed of 1.6% Tris aminomethane, 0.9% citric acid, 1.4% lactose, 80% distilled water, 2.5% raffinose, 20% egg yolk, 0.6% penicillin, and 0.1% streptomycin. Raffinose pentahydrate, citric acid, lactose, and Tris aminomethane were first dissolved and homogenized in 80 mL of distilled water using a magnetic stirrer for 5–10 min. The solution was then cooled from 100 °C to 37 °C, after which penicillin and streptomycin were added and mixed for an additional 15–20 min. Subsequently, 20 mL of this prepared solution was combined with an egg yolk fraction (separated from egg white using filter paper), and homogenized for 20–30 min. The final extender was adjusted pH between 6.7 and 6.9 and stored at 4 °C for 24 h prior to use.

### 4.13. Experimental Design

This study used a completely randomized experimental design with three treatments and three replicates in fresh semen samples. Three treatments were prepared as follows: T1 = a tris-based egg yolk extender (control); T2 = a tris-based egg yolk extender added with vitamin E acetate of 1.5 mg/mL; T3 = a tris-based egg yolk extender added with Phlai extract of 0.63 mg/mL; T4 = a tris-based egg yolk extender added with Njui extract of 0.63 mg/mL; and T5 = a tris-based egg yolk extender added with the mixture of Phlai and Njui extracts of 0.31 mg/mL. All treatments were incubated at 4–5 °C for 0, 1, 3, 6, 24, 48, and 72 h.

### 4.14. Sperm Motility Evaluation

The sperm quality of each treatment at 0, 1, 3, 6, 24, 48, and 72 h was evaluated using AndroVision^®^ software (Minitube of America—MOFA^®^, Verona, WI, USA) connected to a Zeiss AxioScope with a heated stage set at 37 °C (Carl Zeiss MicroImaging GmbH, Göttingen, Germany). Prior to immediate CASA analysis, 5.5 μL aliquots of semen were deposited on prewarmed slides and covered with a coverslip (Hamilton 2X-CEL^®^, Beverly, MA, USA), and subsequently, immediate CASA assessment was conducted. Observations with a 200*×* microscope objective were performed to evaluate sperm motility in four fields. This study examined and statistically analyzed the following parameters: total sperm motility (TM), progressive sperm motility (PM), progressive fast sperm motility (PFM), progressive slow sperm motility (PSM), progressive circular sperm motility (PCM), local sperm motility (LM), and sperm immotility (IM).

### 4.15. Sperm Reactive Oxygen Species (ROS) Production Assessment

The antioxidant activity of Phlai extract on reactive oxygen species (ROS) generation in sperm at 24, 48, and 72 h was evaluated using the 2′,7′-dichlorodihydrofluorescein diacetate (DCFH-DA) assay and double-stained with 4′,6-Diamidino-2-Phenylindole, Dihydrochloride (DAPI). After incubation, 100 µL of sperm suspension from each treatment was placed into a 96-well plate, followed by the addition of 10 µL of 20 µM DCFH-DA. The samples were then incubated at 37 °C in a 5% CO_2_ atmosphere for 30 min in darkness, followed by the addition of 10 µL of DAPI (1 µg/mL) and incubation for 5 min. ROS levels were determined by measuring fluorescence intensity with excitation and emission wavelengths of 488 nm and 617 nm, respectively, using a microplate reader. Additionally, sperm morphology was examined under a fluorescence microscope (Zeiss, Oberkochen, Germany) at 400× magnification.

### 4.16. Statistical Analysis

Experimental data are presented as mean ± standard error of mean (SEM). The Kolmogorov–Smirnov test was used to assess normality. All parameters were analyzed by one-way ANOVA, followed by Duncan’s post hoc test, to compare group means. All experiments were performed in triplicate, and statistical significance was set at *p* < 0.05.

## 5. Conclusions

In conclusion, Phlai and Njui extracts are rich in polyphenols and have antioxidant capacities in cell-free systems. The Njui extract had the highest total phenolics, total tannin, and lycopene content and abundant levels of gallic acid, chlorogenic acid, ellagic acid, and rutin. However, the Phlai extract had the highest total flavonoids and showed a higher concentration of capsaicin, apigenin, kaempferol, and quercetin. Thus, the combined extract was a better choice because it had all the right chemicals in the right amounts. Moreover, the Phlai, Njui, and their combination enhanced sperm motility, especially progressive sperm motility related to the successful rate of fertility. From the results, it may be suggested that the use of Phlai and Njui extracts, particularly in combination, may enhance semen preservation efficiency and add value to agricultural byproducts. These plant extracts may be a good alternative substance for use as a supplement in human sperm preservation procedures, and this should be further studied.

## Figures and Tables

**Table 1:** Total phenolic, total tannins, total flavonoid, total monomeric anthocyanins, and lycopene content of Phlai and Njui extracts.

Sample	Total Phenolic (µg GAE/g Plant Extract)	Total Tannin (µg TAE/g Plant Extract)	Total Flavonoid (µg QE/g Plant Extract)	Total Monomeric Anthocyanins (µg Cyanidin-3-Glucoside E/g Plant Extract)	Lycopene Content (×10^2^ µg/g Plant Extract)
Phlai (*Z. montanum*)	7.30 ± 0.52 ^a^	11.31 ± 1.40 ^a^	7.35 ± 0.99 ^a^	NA	26.82 ± 11.79 ^a^
Njui (*B. ceiba*)	13.68 ± 0.59 ^b^	19.13 ± 0.64 ^b^	2.47 ± 0.21 ^b^	NA	584.95 ± 62.06 ^b^
Phlai and Njui	9.93 ± 0.59 ^c^	14.62 ± 0.65 ^c^	5.25 ± 0.28 ^c^	NA	465.74 ± 44.70 ^b^

The results are presented as the mean value ± standard error of the mean (SEM). All experiments were performed in triplicate, with three independent repetitions. Different superscript letters denote statistically significant differences between groups in each column (*p* < 0.05). All parameters were analyzed by one-way ANOVA followed by Duncan’s test. NA; not detectable.

**Table 2:** The values of capsaicin, gallic acid, chlorogenic acid, ellagic acid, apigenin, kaempferol, rutin, and quercetin of Phlai and Njui extracts.

Compounds	Quantification Wavelength (nm)	Concentration (µg/mg Plant Extract)		
		Phlai (*Z. montanum*)	Njui (*B. ceiba*)	Phlai and Njui
Capsaicin	250	79.54 ± 0.07 ^a^	49.75 ± 0.47 ^b^	54.94 ± 0.06 ^c^
Gallic acid (×10^−2^)	250	0.06 ± 0.01 ^a^	340.25 ± 4.12 ^b^	152.28 ± 0.40 ^c^
Chlorogenic acid	250	0.41 ± 0.01 ^a^	5.21 ± 0.01 ^b^	3.11 ± 0.03 ^c^
Ellagic acid (×10^−2^)	250	4.60 ± 0.37 ^a^	91.47 ± 13.71 ^b^	37.02 ± 0.10 ^c^
Apigenin	250	2.67 ± 0.01 ^a^	NA ^b^	1.44 ± 0.01 ^c^
Kaempferol (×10^−3^)	330	19.10 ± 0.05 ^a^	NA ^b^	13.25 ± 0.03 ^c^
Rutin (×10^−2^)	360	5.17 ± 0.03 ^a^	299.85 ± 13.12 ^b^	134.45 ± 0.65 ^c^
Quercetin (×10^−3^)	360	70.40 ± 0.05 ^a^	NA ^b^	64.10 ± 0.29 ^c^

The results are presented as the mean value ± standard error of the mean (SEM). All experiments were performed in triplicate, with three independent repetitions by HPLC. Quantification was performed at compound-specific wavelengths based on their UV–Vis absorption characteristics: 250 nm for capsaicin, gallic acid, chlorogenic acid, ellagic acid, and apigenin; 330 nm for kaempferol; and 360 nm for rutin and quercetin. Different superscript letters denote statistically significant differences between groups in each row (*p* < 0.05). All parameters were analyzed by one-way ANOVA followed by Duncan’s test. NA; not detectable.

**Table 3:** The proportion of total sperm motility (TM), progressive sperm motility (PM), progressive fast sperm motility (PFM), progressive slow sperm motility (PSM), progressive circular sperm motility (PCM), local sperm motility (LM), and sperm immotility (IM) of extender with Phlai, Njui, and combined extracts compared to normal extender from beginning to 72 h incubation at 4–5 °C.

Time	Treatments	TM (%)	PM (%)	PFM (%)	PSM (%)	PCM (%)	LM (%)	IM (%)
H0	Normal	76.83 ± 7.25	70.43 ± 9.83	44.98 ± 12.99	25.06 ± 4.00	0.36 ± 0.25	6.43 ± 2.61	23.17 ± 7.25
	Vit E	76.56 ± 6.89	67.85 ± 11.19	40.95 ± 14.79	26.55 ± 4.16	0.34 ± 0.34	8.71 ± 4.33	23.44 ± 6.89
	Phlai	76.98 ± 7.10	71.70 ± 8.88	47.26 ± 11.82	24.20 ± 3.83	0.24 ± 0.20	5.28 ± 1.96	23.02 ± 7.10
	Njui	77.56 ± 7.10	71.80 ± 9.38	45.39 ± 11.99	26.15 ± 4.43	0.27 ± 0.18	5.76 ± 2.60	22.44 ± 7.10
	Phlai & Njui	77.56 ± 7.24	72.55 ± 8.86	45.72 ± 13.54	26.42 ± 5.39	0.41 ± 0.41	5.01 ± 1.71	22.44 ± 7.24
H1	Normal	69.56 ± 12.06	66.66 ± 12.53	42.06 ± 8.63	24.20 ± 4.04	0.40 ± 0.30	2.90 ± 1.86	30.44 ± 12.06
	Vit E	71.22 ± 8.16	68.31 ± 8.02	41.03 ± 7.47	27.01 ± 5.04	0.27 ± 0.11	2.92 ± 1.08	28.78 ± 8.16
	Phlai	71.88 ± 8.28	67.14 ± 9.36	43.46 ± 7.53	23.08 ± 4.25	0.60 ± 0.30	4.74 ± 1.35	28.12 ± 8.28
	Njui	72.21 ± 7.92	68.13 ± 8.66	44.99 ± 9.27	22.57 ± 1.10	0.56 ± 0.27	4.08 ± 1.58	27.79 ± 7.92
	Phlai & Njui	71.00 ± 8.27	66.83 ± 9.05	46.99 ± 9.97	19.41 ± 2.38	0.42 ± 0.10	4.18 ± 0.78	29.00 ± 8.27
H3	Normal	63.71 ± 7.44	59.40 ± 7.77	33.80 ± 5.24	25.54 ± 2.58	0.06 ± 0.06	4.31 ± 0.89	36.29 ± 7.44
	Vit E	65.46 ± 7.93	61.32 ± 7.91	38.61 ± 4.68	22.24 ± 3.41	0.47 ± 0.19	4.14 ± 0.62	34.54 ± 7.93
	Phlai	64.40 ± 7.36	59.32 ± 8.57	40.15 ± 6.06	18.92 ± 3.27	0.25 ± 0.11	5.08 ± 1.21	35.60 ± 7.36
	Njui	65.30 ± 7.58	59.78 ± 8.25	36.57 ± 5.65	22.99 ± 3.09	0.21 ± 0.13	5.52 ± 1.43	34.70 ± 7.58
	Phlai & Njui	64.65 ± 7.33	61.66 ± 8.07	37.65 ± 5.56	23.87 ± 5.46	0.14 ± 0.07	2.99 ± 0.92	35.35 ± 7.33
H6	Normal	62.30 ± 7.77	58.08 ± 7.21	35.30 ± 3.73	22.71 ± 3.51	0.07 ± 0.04	4.22 ± 0.63	37.70 ± 7.77
	Vit E	63.28 ± 8.05	60.89 ± 7.75	38.12 ± 4.98	22.30 ± 5.26	0.47 ± 0.25	2.39 ± 0.63	36.72 ± 8.05
	Phlai	62.90 ± 7.67	58.72 ± 7.48	40.32 ± 6.89	18.33 ± 0.62	0.08 ± 0.08	4.17 ± 0.37	37.10 ± 7.67
	Njui	64.96 ± 7.93	61.78 ± 7.62	38.84 ± 6.61	22.54 ± 0.93	0.04 ± 0.04	2.36 ± 1.36	35.04 ± 7.93
	Phlai & Njui	63.65 ± 7.57	60.36 ± 7.69	41.70 ± 6.64	18.15 ± 0.87	0.52 ± 0.18	3.28 ± 0.48	36.35 ± 7.57
H24	Normal	46.65 ± 2.71	40.87 ± 3.00	25.91 ± 2.59	14.97 ± 0.41 ^b^	0.00 ± 0.00	5.77 ± 0.29 ^a^	53.35 ± 2.71
	Vit E	58.57 ± 6.80	55.30 ± 6.58	37.43 ± 5.66	17.74 ± 1.01 ^ab^	0.14 ± 0.07	3.27 ± 0.33 ^b^	41.39 ± 6.81
	Phlai	59.69 ± 7.15	56.33 ± 6.90	39.06 ± 5.02	17.15 ± 2.64 ^ab^	0.12 ± 0.07	3.36 ± 0.29 ^b^	40.31 ± 7.15
	Njui	62.69 ± 7.13	58.35 ± 7.03	36.47 ± 6.92	21.67 ± 1.80 ^a^	0.21 ± 0.11	4.34 ± 0.11 ^ab^	37.31 ± 7.13
	Phlai & Njui	61.93 ± 7.21	57.49 ± 8.19	39.09 ± 7.95	18.29 ± 2.54 ^ab^	0.10 ± 0.05	4.45 ± 1.08 ^ab^	38.07 ± 7.21
H48	Normal	36.05 ± 1.97	34.41 ± 1.67 ^b^	22.41 ± 3.82	11.85 ± 5.57	0.15 ± 0.07	1.65 ± 0.30	63.95 ± 1.97 ^a^
	Vit E	44.99 ± 4.49	41.77 ± 3.47 ^ab^	29.02 ± 3.26	12.45 ± 4.20	0.30 ± 0.30	3.22 ± 1.22	55.01 ± 4.49 ^ab^
	Phlai	46.31 ± 4.44	40.70 ± 2.29 ^ab^	28.62 ± 1.75	12.05 ± 3.80	0.03 ± 0.03	5.61 ± 2.17	53.69 ± 4.44 ^ab^
	Njui	48.58 ± 5.49	43.92 ± 4.25 ^ab^	24.98 ± 2.00	18.38 ± 4.14	0.56 ± 0.52	4.66 ± 1.50	51.42 ± 5.49 ^ab^
	Phlai & Njui	49.61 ± 3.93	46.20 ± 3.17 ^a^	25.53 ± 3.35	20.64 ± 0.21	0.03 ± 0.03	3.41 ± 0.75	50.39 ± 3.93 ^b^
H72	Normal	19.44 ± 7.93 ^b^	17.75 ± 6.96 ^b^	9.72 ± 3.25	8.03 ± 3.71 ^b^	0.00 ± 0.00	1.68 ± 0.96 ^b^	80.56 ± 7.93 ^a^
	Vit E	32.82 ± 10.49 ^ab^	29.12 ± 9.92 ^ab^	15.36 ± 5.53	13.76 ± 4.39 ^ab^	0.00 ± 0.00	3.70 ± 0.57 ^ab^	67.18 ± 10.49 ^ab^
	Phlai	42.75 ± 2.96 ^a^	38.38 ± 1.91 ^a^	19.73 ± 2.87	18.58 ± 0.95 ^ac^	0.00 ± 0.00	4.45 ± 1.05 ^a^	57.25 ± 2.96 ^b^
	Njui	44.88 ± 2.95 ^a^	40.16 ± 2.36 ^a^	12.53 ± 4.85	27.60 ± 2.52 ^a^	0.04 ± 0.04	4.72 ± 0.59 ^a^	55.12 ± 2.95 ^b^
	Phlai & Njui	43.32 ± 2.85 ^a^	39.04 ± 2.83 ^a^	16.49 ± 2.53	22.55 ± 0.30 ^ac^	0.00 ± 0.00	4.28 ± 0.02 ^a^	56.68 ± 2.85 ^b^

The results are presented as the mean value ± standard error of the mean (SEM). All experiments were performed in triplicate (n = 3), with three independent repetitions. Different superscript letters denote statistically significant differences between groups in each column (*p* < 0.05). All parameters were analyzed by one-way ANOVA followed by Duncan’s test.

## Data Availability

Data are contained within the article and Supplementary Materials.

## Supplementary Materials

The following supporting information can be downloaded at: https://www.mdpi.com/article/10.3390/molecules31020368/s1. Figure S1: HPLC chromatograms of phytochemical compounds in Phlai and Njui extracts.
