# Supplementary material for: Influence of Phlai (Zingiber montanum) and Njui (Bombax ceiba) Extracts in Bull Semen Extender on Antioxidant Activity and Sperm Quality
Source: Molecules. 2026 Jan 20;31(2):368. doi: 10.3390/molecules31020368 (PMC12844420; doi:10.3390/molecules31020368)
Supplement: Supplementary file 1 [file molecules-31-00368-s001.zip › molecules-4029089-supplementary.pdf]

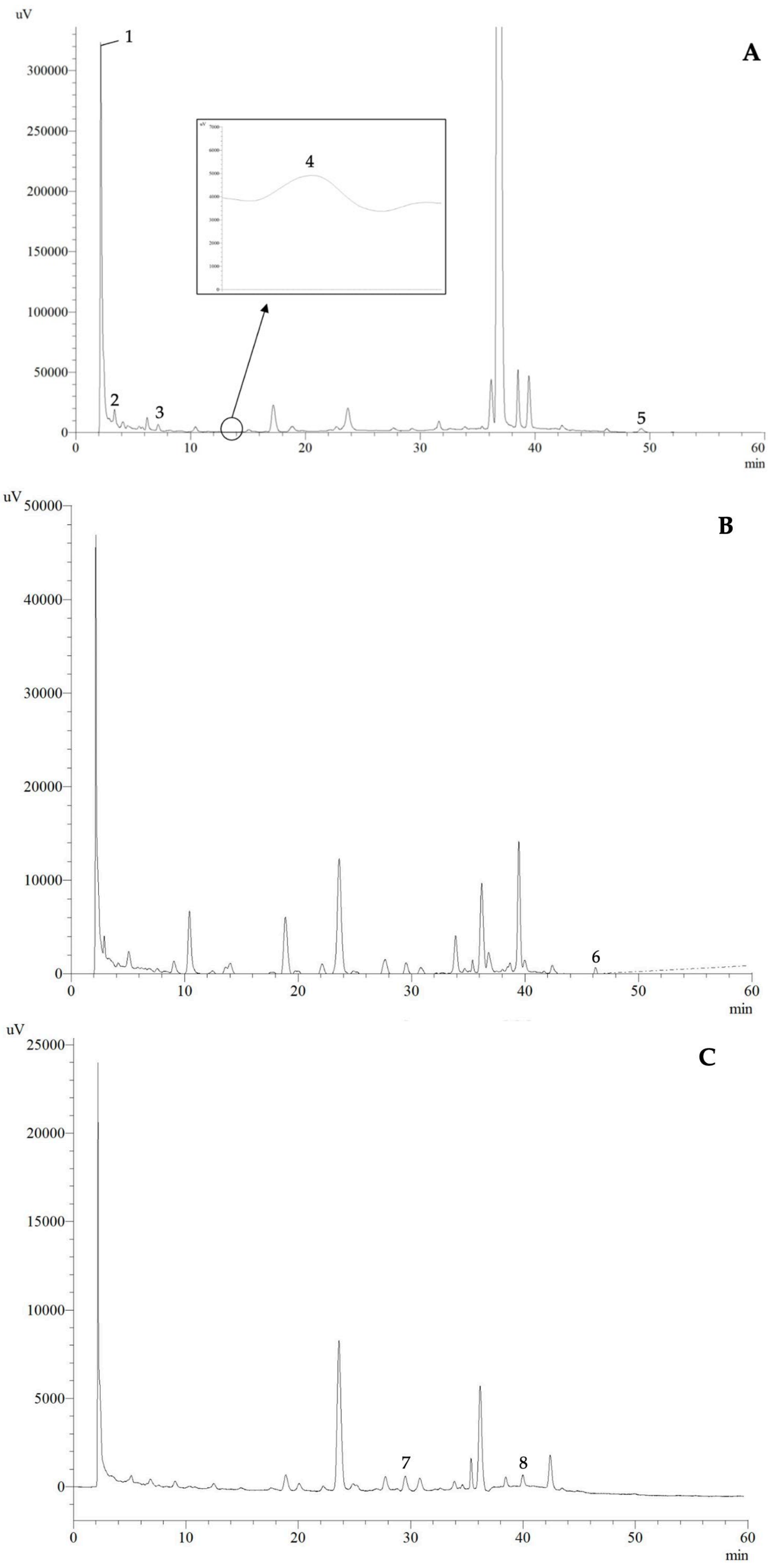

A
uV
min
1
2
3
4
5
B
6
C
7
8

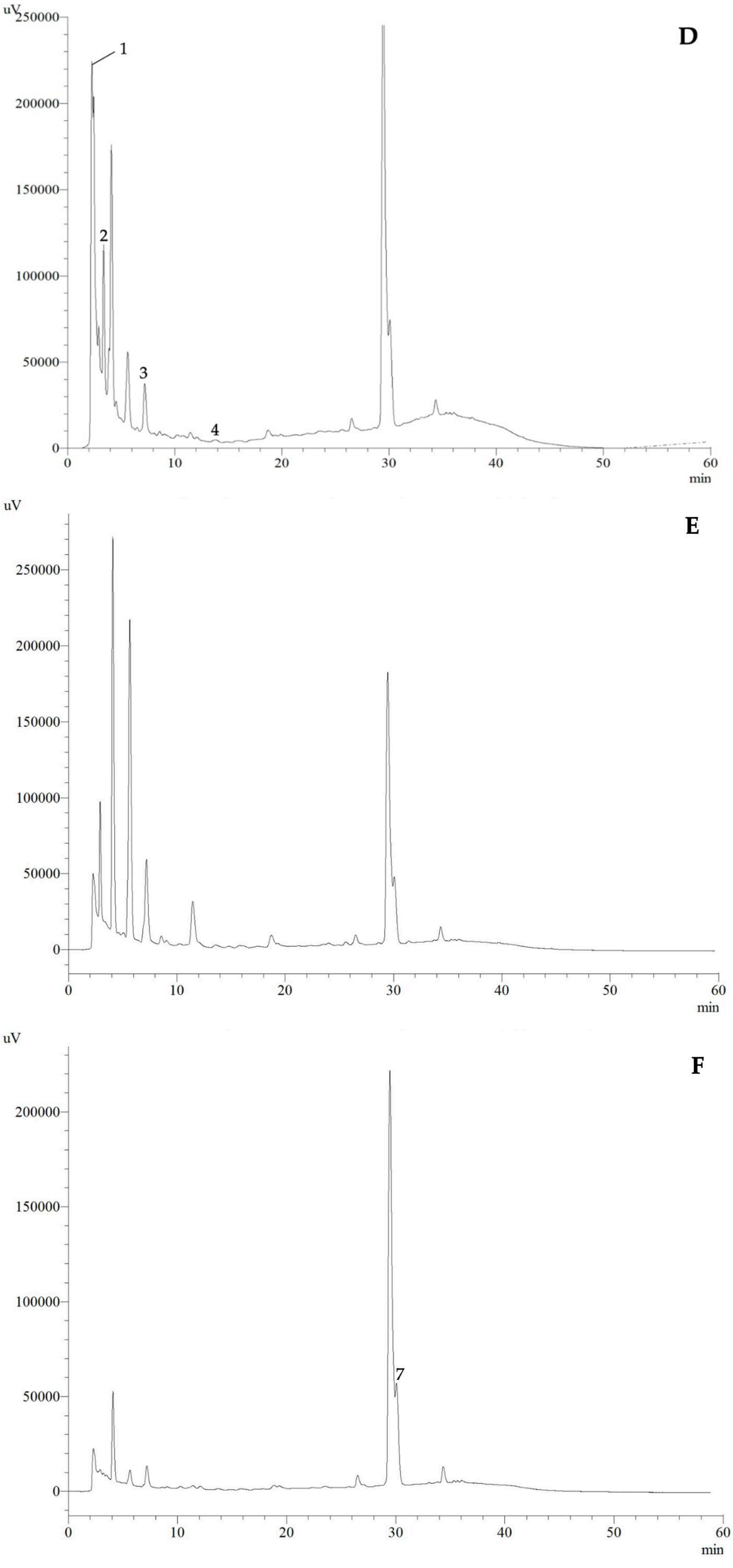

D
uV
250000
200000
150000
100000
50000
0
1
2
3
4
0
10
20
30
40
50
60
min
E
uV
250000
200000
150000
100000
50000
0
0
10
20
30
40
50
60
min
F
uV
200000
150000
100000
50000
0
7
0
10
20
30
40
50
60
min

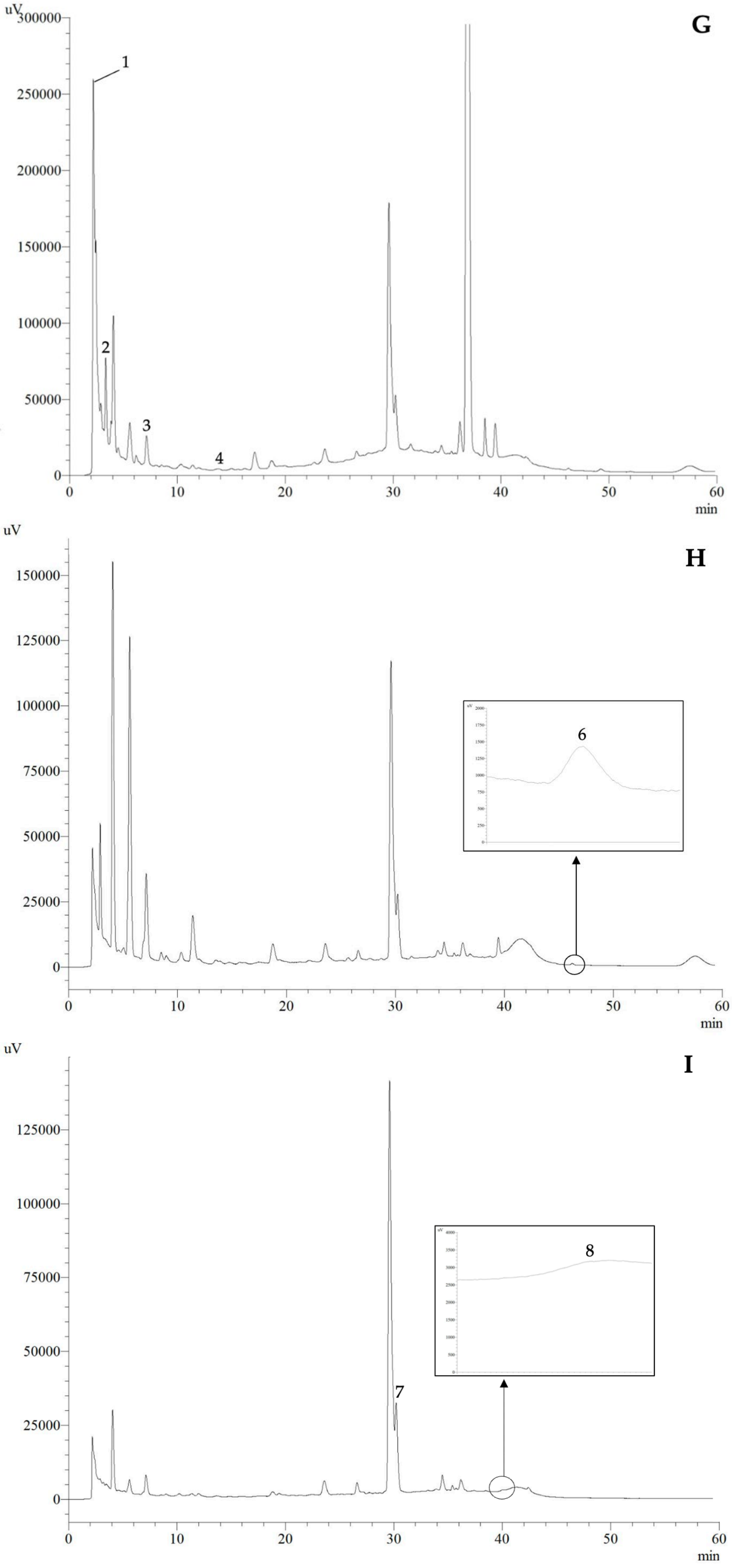

G
uV
300000
250000
200000
150000
100000
50000
0
1
2
3
4
0
10
20
30
40
50
60
min
H
uV
150000
125000
100000
75000
50000
25000
0
6
0
10
20
30
40
50
60
min
I
uV
125000
100000
75000
50000
25000
0
7
8
0
10
20
30
40
50
60
min

Figure S1. HPLC chromatograms of phytochemical compounds shown in the Phlai (**A-C**), the Njui (**D-F**), and the Phlai and Njui (**G-I**) from Column, Purospher® Star PR-18; mobile phase, 0.1% formic acid in water and 8% acetonitrile; flow rate, 0.8 mL/min; detection wavelength, 250 nm (**A, D, and G**), 330 (**B, E, and H**), and 360nm (**C, F and I**). Peak identification: peak 1, capsaicin; peak 2, gallic acid; peak 3, chlorogenic acid; peak 4, ellagic acid; peak 5, apigenin; peak 6, kaempferol; peak 7, rutin; and peak 8, quercetin.
